# Glucagon-Like Peptide-1 Receptor Agonists and Reproductive Health: A Narrative Review for Obstetrician-Gynecologists

**DOI:** 10.7759/cureus.111915

**Published:** 2026-07-01

**Authors:** Sara Almaradweh, Emad Mousa

**Affiliations:** 1 Medicine, Avalon University School of Medicine, Willemstad, CUW; 2 Obstetrics and Gynecology, Logan Regional Hospital, Logan, USA

**Keywords:** contraception, glp-1 receptor agonists, lactation, obesity, obstetrics and gynecology, polycystic ovary syndrome, preconception counseling, pregnancy, reproductive health, type 2 diabetes

## Abstract

The expanding use of glucagon-like peptide-1 receptor agonists (GLP-1 RAs) and dual glucose-dependent insulinotropic polypeptide (GIP)/GLP-1 RAs for obesity and type 2 diabetes has important implications for reproductive-aged patients. As these medications are increasingly prescribed before conception and after delivery, obstetrician-gynecologists need practical guidance on contraception, pregnancy planning, inadvertent pregnancy exposure, medication discontinuation, gestational weight trajectory, postpartum use, and lactation. This narrative review synthesizes current evidence on GLP-1 RA and dual GIP/GLP-1 RA use across the reproductive spectrum, including mechanisms relevant to ovulation and polycystic ovary syndrome (PCOS), pharmacokinetic interactions with oral hormonal contraceptives, animal and human pregnancy safety data, preconception washout recommendations, management after inadvertent early-pregnancy exposure, postpartum prescribing, breastfeeding considerations, and unresolved research gaps. Available evidence suggests potential reproductive benefits in some patients with metabolic dysfunction or PCOS, but current pregnancy and lactation data remain limited. Until stronger prospective safety data are available, counseling should be individualized, agent-specific, and centered on pregnancy intention, contraceptive reliability, metabolic risk, and shared decision-making.

## Introduction and background

The use of semaglutide, tirzepatide, and related incretin-based therapies has expanded rapidly for obesity, type 2 diabetes, and weight-related metabolic disease. Glucagon-like peptide-1 receptor agonists (GLP-1 RAs) have demonstrated substantial reductions in body weight in phase three trials, with tirzepatide showing greater efficacy than semaglutide [1]. As prescribing expands beyond diabetes management, reproductive counseling has become increasingly relevant for reproductive-aged women. As of 2024, GLP-1 RA prescribing among pregnant patients in the United States increased from 0.2 to 6.4 per 1,000 deliveries in the predelivery period and from 0.3 to 14.6 per 1,000 deliveries postdelivery [2]. These trends highlight the growing importance of understanding the reproductive implications of these agents.

Approximately 40% of pregnancies in the United States remain unintended, and patients using GLP-1 RAs may experience weight loss and metabolic improvement that can restore ovulatory function and lead to unexpected conception. This phenomenon has been described in public and clinical discussions as “Ozempic babies” [3,4]. At the same time, important clinical questions remain regarding contraceptive reliability, timing of medication discontinuation, inadvertent early-pregnancy exposure, gestational weight trajectory after discontinuation, postpartum prescribing, and lactation safety. Practical guidance for obstetrician-gynecologists remains limited because available evidence is distributed across endocrinology, obesity medicine, reproductive medicine, pregnancy safety data, pharmacology, and lactation literature. Existing recommendations also vary by agent and clinical context. This narrative review synthesizes current evidence and provides a practical counseling framework for clinicians caring for reproductive-aged patients using GLP-1 RAs or dual glucose-dependent insulinotropic polypeptide (GIP)/GLP-1 RAs.

## Review

Literature search and selection approach

This narrative review was developed through targeted searches of PubMed, Google Scholar, regulatory labeling, and contemporary clinical guidance relevant to GLP-1 RAs, dual GIP/GLP-1 RAs, and reproductive health. Sources were selected to prioritize recent evidence, clinical relevance, and applicability to obstetrician-gynecologists, with emphasis on literature published through 2026. The review included studies and guidance addressing mechanisms of action, pharmacokinetics, ovulation and polycystic ovary syndrome (PCOS), contraceptive interactions, pregnancy exposure, preconception counseling, lactation, postpartum use, and unresolved clinical questions.

This article was designed as a narrative review rather than a systematic or scoping review; therefore, Preferred Reporting Items for Systematic Reviews and Meta-Analyses (PRISMA) methodology, formal study screening, risk-of-bias assessment, and meta-analysis were not performed. Because the goal was to provide a clinically oriented narrative synthesis for obstetrician-gynecologists, sources were selected for relevance to reproductive counseling, pregnancy planning, contraception, lactation, and postpartum care rather than through prespecified systematic-review eligibility criteria.

Because of heterogeneity in study designs, patient populations, exposure windows, and outcomes, no quantitative pooling, meta-analysis, meta-regression, or formal certainty-of-evidence grading was performed.

Mechanism of action and pharmacokinetics relevant to reproduction

GLP-1 is an incretin hormone released from intestinal L cells after nutrient intake. In its native form, GLP-1 is rapidly inactivated by dipeptidyl peptidase-4, resulting in a very short circulating half-life of approximately two to three minutes [5]. Therapeutic GLP-1 RAs were developed to prolong GLP-1 activity through structural modifications that reduce enzymatic breakdown, including amino acid substitutions, fatty acid side chains, albumin binding, and fusion with immunoglobulin Fc domains [5,6].

The clinically used agents differ meaningfully in dosing frequency and persistence in circulation. Liraglutide is given as a once-daily subcutaneous injection and has a half-life of approximately 13 hours. Semaglutide can be administered once weekly by subcutaneous injection or once daily by the oral route; its half-life is approximately 155 to 184 hours, and steady-state concentrations are generally reached after four to five weeks [7,8]. Dulaglutide is administered once weekly and has a half-life of approximately 120 hours. Tirzepatide, a dual GIP/GLP-1 RA, is also administered once weekly and has a half-life of approximately 120 hours [7].

The major pharmacologic actions of these medications include glucose-dependent stimulation of insulin secretion, inhibition of glucagon release, slowing of gastric emptying, and regulation of appetite through central pathways [6,9]. These effects are directly relevant to reproductive care because they influence weight, glycemic control, oral medication absorption, and the timing of medication discontinuation before conception.

GLP-1 signaling is also biologically relevant within the reproductive system. GLP-1 receptors have been identified in several components of the reproductive axis, including the hypothalamus, pituitary, ovaries, endometrium, and placenta [3,10]. Experimental studies suggest that GLP-1-related pathways may influence gonadotropin-releasing hormone and luteinizing hormone (LH) activity through hypothalamic mechanisms [9]. Additional preclinical work suggests possible ovarian effects beyond weight loss, including effects on follicular development mediated through reduced CXCL10 secretion from granulosa cells and suppression of JAK2 phosphorylation [9]. In animal models, semaglutide has also been associated with improvement in obesity-related reproductive dysfunction through effects on inflammatory and metabolic pathways involving SIRT-associated signaling [11].

Because semaglutide and tirzepatide remain in circulation for prolonged periods, reproductive counseling should account for persistent drug exposure after discontinuation. This is especially important when discussing preconception planning, unintended pregnancy exposure, and agent-specific washout recommendations.

Effects on ovulation, menstrual cyclicity, and PCOS

PCOS is a frequent cause of anovulatory infertility, particularly among patients with overweight or obesity [12]. Insulin resistance, compensatory hyperinsulinemia, altered gonadotropin signaling, and excess ovarian androgen production are central features of PCOS, which makes incretin-based therapy biologically relevant in this population [9,12].

In a 2026 systematic review and meta-analysis of randomized controlled trials, liraglutide was evaluated in women with PCOS and overweight or obesity. Compared with metformin or placebo, liraglutide was associated with greater improvements in BMI, insulin resistance (as measured by the homeostatic model assessment of insulin resistance), and LH levels [9]. Liraglutide was also associated with modest improvements in menstrual frequency and higher sex hormone-binding globulin concentrations [9]. These effects are likely mediated by both metabolic improvement, including reduced hyperinsulinemia and decreased ovarian androgen production, and possible direct effects on the hypothalamic-pituitary-ovarian axis [9].

A broader meta-analysis of 11 randomized controlled trials, including 840 women with PCOS, reported that GLP-1 RAs improved natural pregnancy rates and menstrual cyclicity compared with metformin [13]. Reported outcomes included a relative risk of 1.72 for natural pregnancy and improved menstrual regularity with a standardized mean difference of 1.72 [13]. Additionally, a pilot randomized trial found that low-dose liraglutide combined with metformin improved in vitro fertilization pregnancy rates among women with obesity and PCOS who had previously responded poorly to fertility treatment, despite similar weight loss between groups [9].

Animal fertility data also suggest potential reproductive effects. In female rats, semaglutide exposure was associated with longer estrus cycles across dose levels and a small decrease in corpora lutea at doses of 0.7-fold or greater relative to the maximum recommended human dose. These findings were interpreted as likely adaptive responses to reduced food intake and body weight rather than clear evidence of direct ovarian toxicity [14].

Clinically, these findings suggest that GLP-1 RAs may improve ovulatory function in some previously anovulatory patients with PCOS. This potential benefit also presents an important counseling challenge: patients who previously believed pregnancy was unlikely may become at risk for unintended pregnancy and should receive proactive contraception and preconception counseling.

Drug interactions with hormonal contraceptives

Because GLP-1 RAs slow gastric emptying, they can affect the rate at which some orally administered medications are absorbed. This is particularly important for oral hormonal contraceptives, where reduced or delayed absorption could theoretically affect contraceptive reliability [6,15]. The magnitude of this interaction, however, differs across agents.

A systematic review evaluating pharmacokinetic interactions between GLP-1 RAs and oral medications found that these agents may lower peak drug concentration (Cmax) and prolong time to peak concentration (tmax) for certain oral drugs, including oral contraceptives [15]. Despite these changes, total drug exposure, reflected by the area under the concentration-time curve (AUC), was generally preserved and was not considered clinically meaningful for most oral medications [15]. Therefore, routine dose adjustment is usually not required when most GLP-1 RAs are used with oral therapies [15].

Tirzepatide is the main exception. As a dual GIP/GLP-1 RA, tirzepatide has a stronger effect on gastric emptying early in treatment, especially after initiation and dose escalation, before tachyphylaxis develops [16]. Pharmacokinetic studies have shown reduced oral contraceptive exposure when tirzepatide is co-administered with combined oral contraceptives, including reductions in AUC and Cmax and delayed tmax [16]. By contrast, available studies of semaglutide, liraglutide, exenatide, and dulaglutide have not shown clinically significant reductions in oral contraceptive exposure [16]. A summary of pharmacokinetic interactions between GLP-1 RAs, tirzepatide, and oral hormonal contraceptives is provided in Table 1.

**Table 1:** Summary of pharmacokinetic interactions between GLP-1 RAs and oral hormonal contraceptives This table compares the effects of GLP-1 RAs and dual GIP/GLP-1 RA therapy on combined oral contraceptive pharmacokinetics. Most GLP-1 RAs may delay time to maximum concentration and/or reduce peak concentration without clinically meaningful changes in overall oral contraceptive exposure. Tirzepatide produces a clinically significant interaction during initiation and dose escalation, and backup or non-oral contraception is recommended during this period. Data derived from pharmacokinetic reviews, contraceptive interaction studies, and pharmacokinetic modeling studies [6,15-17]. AUC: area under the concentration-time curve, Cmax: maximum plasma concentration, tmax: time to maximum concentration, EE: ethinylestradiol, LNG: levonorgestrel, OC: oral contraceptive, SC: subcutaneous, IR: immediate release, ER: extended release, LARC: long-acting reversible contraception, GLP-1 RA: glucagon-like peptide-1 receptor agonist, GIP: glucose-dependent insulinotropic polypeptide

Agent	Class	Half-life	Effect on OC AUC	Effect on OC Cmax	Effect on OC tmax	Clinical significance	Manufacturer/guideline recommendation
Semaglutide (SC)	GLP-1 RA	~7 days (168 h)	No significant change (EE ratio 1.06 (90% CI 0.99-1.13); LNG ratio 1.06 (0.97–1.17))	↓ 12% EE, ↓ 13% LNG	Delayed ~1.5 h	Not clinically significant	No backup contraception required
Oral semaglutide	GLP-1 RA	~7 days	No significant change (EE AUC ratio 1.06 (1.01-1.10); LNG 1.06 (0.97-1.17))	Not significantly affected	Not significantly affected	Not clinically significant	No backup contraception required
Liraglutide	GLP-1 RA	~13 h	No significant change (EE AUC ratio 1.06 (0.99-1.13); LNG AUC ratio 1.18 (1.04-1.34))	↓ 12% EE, ↓ 13% LNG	Delayed ~1.5 h	Not clinically significant	No backup contraception required
Dulaglutide	GLP-1 RA	~5 days	No significant change	Minimal reduction	Minimal delay	Not clinically significant	No backup contraception required
Exenatide	GLP-1 RA	~2.4 h (IR); ~2 weeks (ER)	No significant change	↓ Cmax with IR formulation	Delayed with IR	Not clinically significant	No backup contraception required
Tirzepatide	Dual GIP/GLP-1 RA	~5 days (117 h)	Statistically significant ↓ in AUC during initiation/dose escalation	Significant ↓ Cmax	Significant delay	Clinically significant during initiation and dose escalation; effect diminishes at steady state	Use backup contraception, barrier method, or LARC until the maintenance dose has been achieved and used for ≥4 weeks

Because of this interaction, patients using oral hormonal contraceptives should be counseled to use an additional barrier method or switch to a non-oral contraceptive method during tirzepatide initiation and dose escalation [16]. This interaction does not apply to contraceptive methods that bypass gastrointestinal absorption, including intrauterine devices, implants, injectable progestins, transdermal patches, and vaginal rings.

A comprehensive pharmacokinetic review similarly identified oral contraceptives with tirzepatide and levothyroxine with oral semaglutide as clinically serious situations in which exposure changes may warrant additional attention [6]. Physiologically based pharmacokinetic modeling also supports that GLP-1 RA-induced delays in gastrointestinal motility can alter oral medication pharmacokinetics, although the clinical relevance depends on the medication involved [17].

For clinical practice, the most prudent approach for reproductive-aged women using GLP-1 RAs, particularly tirzepatide, is to discuss contraceptive efficacy proactively. Long-acting reversible contraception methods that do not depend on gastrointestinal absorption may be preferred, and patients using oral hormonal contraceptives should be counseled about backup contraception during tirzepatide initiation and dose escalation.

Pregnancy safety data: animal and human evidence

Animal Data

Animal reproductive toxicology studies have raised concerns about the use of GLP-1 RAs during pregnancy. In rat studies of semaglutide exposure during organogenesis, fetal loss, structural abnormalities, and growth effects were reported at maternal exposures considered clinically relevant [14,18]. Rabbit and cynomolgus monkey studies also reported early pregnancy loss and structural abnormalities, with effects seen at clinical exposure levels in rabbits and at exposures at least two times the maximum recommended human dose in monkeys [14]. Interpretation of these findings is complicated by the fact that maternal weight loss occurred in the same studies, making it difficult to separate possible direct drug effects from fetal effects related to reduced maternal nutrition or weight loss [19,20].

A systematic review of preclinical data identified recurring lower fetal weight or impaired growth, delayed ossification, and skeletal variants with GLP-1 RA exposure, most often in the setting of reduced maternal food intake and lower maternal weight gain [19]. Available placental transfer data are limited. Exendin-4 did not appear to cross the maternal-fetal placental interface in mouse models, and limited human data with liraglutide did not show meaningful maternal-to-fetal transfer [3,19].

Human Data

Human pregnancy data remain limited, but current findings are less concerning than those from animal studies. A 2026 systematic review of 36 studies reported that observational cohorts have not shown a consistent association between periconceptional or early pregnancy exposure to GLP-1-based therapies and major congenital malformations, fetal growth restriction, stillbirth, or neonatal mortality when compared with insulin-treated or disease-matched groups [20].

Pregnancy outcomes from semaglutide clinical development programs have been described. Across 20 trials, including 5,936 female participants exposed to semaglutide, 40 pregnancies occurred in the semaglutide group. Outcomes included 22 healthy live births, six spontaneous abortions, seven elective terminations, one congenital abnormality, and one ectopic pregnancy. Among 12 pregnancies in the placebo group, outcomes included five healthy live births, two congenital abnormalities, two elective terminations, one spontaneous abortion, and one stillbirth [7].

A systematic review focused on semaglutide exposure included five studies and 1,128 exposed pregnancies. The review did not identify a clear signal for increased birth defects. However, one included study reported an 8.3% prevalence of congenital malformations without a statistically significant increase compared with insulin exposure [21]. The reported spontaneous abortion rate of 23% was similar to rates seen in diabetes and obesity reference populations [21].

A large observational population-based cohort study of 938 pregnancies among patients with type 2 diabetes compared periconceptional GLP-1 RA exposure with insulin exposure and found no statistically significant increase in the risk of major congenital malformations [4]. However, this study lacked information on maternal glycemic control, which is an important limitation because maternal hyperglycemia is a major contributor to congenital anomaly risk in pregnancies complicated by diabetes [4].

Despite these preliminary reassurances, the available evidence remains insufficient to definitively establish safety. Current human studies are observational and limited by residual confounding, small exposed sample sizes, variable exposure windows, and heterogeneous patient populations [3,20].

Periconception counseling and recommended washout periods

Current product labeling and clinical guidelines generally recommend discontinuation of GLP-1 RAs before pregnancy, although the specific timing varies by agent and source. The FDA labeling for semaglutide recommends stopping therapy at least two months before a planned pregnancy, reflecting its prolonged elimination period [8,18]. Recent guidelines, reviews, and observational studies emphasize that counseling should address pregnancy outcomes after GLP-1 RA exposure or discontinuation, preconception counseling, gestational weight trajectory, postpartum prescribing trends, and long-term cardiometabolic follow-up [22-28]. For tirzepatide, the Canadian product monograph recommends discontinuation at least one month before planned conception [29]. The U.S. prescribing information for tirzepatide does not provide a specific preconception washout interval [30].

The American Diabetes Association (ADA) Standards of Care recommend discontinuing GLP-1 RAs and dual GIP/GLP-1 RAs before pregnancy and using contraception while these medications are being taken [22]. The ADA also notes that several months may be needed to allow medication clearance, transition to pregnancy-compatible therapy, and optimization of preconception glycemic targets [22]. Patients should be counseled that stopping therapy may be followed by weight recurrence or worsening glycemic control [22].

**Figure 1:**
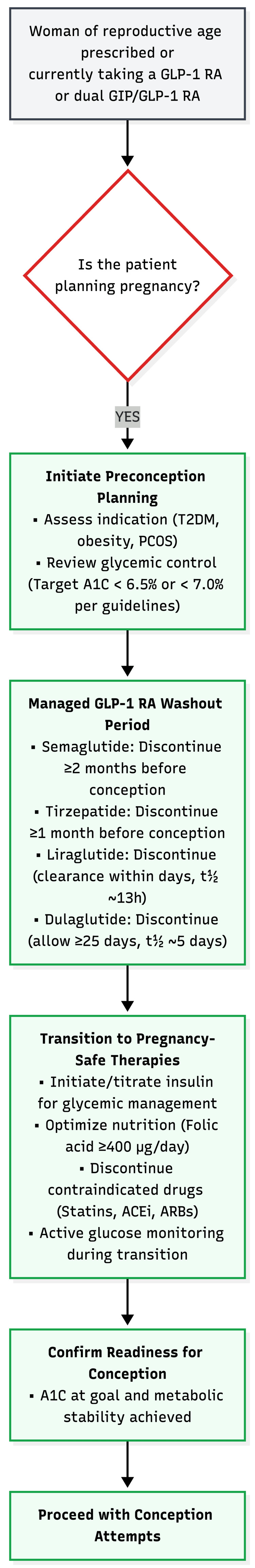
Preconception counseling and washout algorithm for reproductive-aged women receiving GLP-1 RAs or dual GIP/GLP-1 RAs This clinical decision flowchart outlines management for reproductive-aged patients planning pregnancy while receiving GLP-1 RAs or dual GIP/GLP-1 RAs. The algorithm emphasizes preconception assessment, agent-specific discontinuation/washout, transition to pregnancy-safe therapies, metabolic stabilization, and confirmation of readiness before attempts at conception. Washout intervals are based on pharmacokinetic half-lives, prescribing information, and current clinical guidance. Data derived from the Endocrine Society/European Society of Endocrinology Clinical Practice Guideline [3], FDA/product labeling for semaglutide [18], ADA Standards of Care 2026 [22], Canadian tirzepatide prescribing information [29], and U.S. tirzepatide prescribing information [30]. GLP-1 RA: glucagon-like peptide-1 receptor agonist, GIP: glucose-dependent insulinotropic polypeptide, T2DM: type 2 diabetes mellitus, PCOS: polycystic ovary syndrome, A1C: glycated hemoglobin, ACEi: angiotensin-converting enzyme inhibitor, ARBs: angiotensin receptor blockers, FDA: Food and Drug Administration, ADA: American Diabetes Association

The Endocrine Society and European Society of Endocrinology Joint Clinical Practice Guideline recommends discontinuing GLP-1 RA therapy before conception in individuals with type 2 diabetes, transitioning in a timely manner to an alternative antihyperglycemic treatment, and maintaining glycemic control after discontinuation [3]. The guideline emphasizes individualized timing based on the likelihood of conception, the agent being used, and the risks associated with prolonged time off therapy [3]. For patients planning pregnancy while using a GLP-1 RA or dual GIP/GLP-1 RA, a preconception counseling and washout pathway is shown in Figure 1.

**Figure 2:**
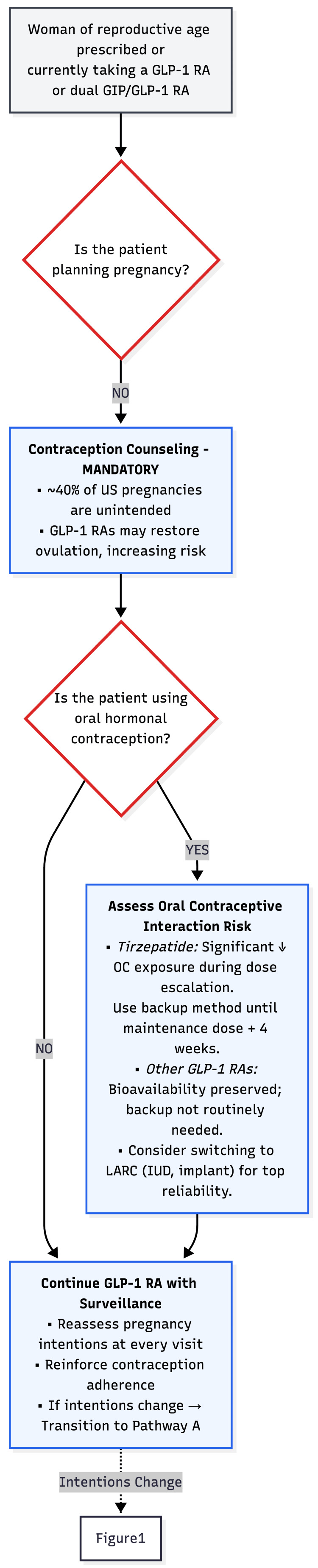
Contraceptive counseling and oral contraceptive interaction algorithm for reproductive-aged women receiving GLP-1 RAs or dual GIP/GLP-1 RAs This clinical decision flowchart outlines management for reproductive-aged patients not currently planning pregnancy while receiving GLP-1 RAs or dual GIP/GLP-1 RAs. The algorithm emphasizes mandatory contraception counseling, assessment of oral hormonal contraceptive use, recognition of the clinically significant interaction between tirzepatide and oral contraceptives during initiation and dose escalation, and continued surveillance with reassessment of pregnancy intentions at follow-up visits. If pregnancy intentions change, patients transition to the preconception planning pathway shown in Figure 1. Data derived from the Endocrine Society/European Society of Endocrinology Clinical Practice Guideline [3], pharmacokinetic reviews and contraceptive interaction studies [6,15,16], ADA Standards of Care 2026 [22], and tirzepatide prescribing information [30]. GLP-1 RA: glucagon-like peptide-1 receptor agonist, GIP: glucose-dependent insulinotropic polypeptide, OC: oral contraceptive, LARC: long-acting reversible contraception, IUD: intrauterine device, ADA: American Diabetes Association

For patients who are not currently planning pregnancy, a contraception counseling and oral contraceptive interaction pathway is shown in Figure 2.

**Figure 3:**
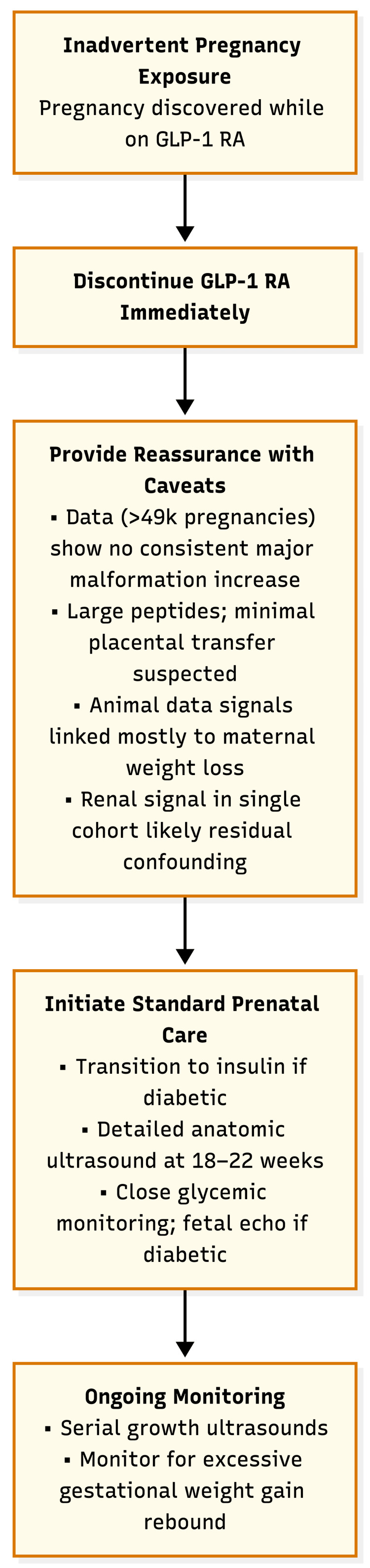
Management of inadvertent pregnancy exposure to GLP-1 RAs This flowchart outlines a practical approach when pregnancy is discovered during treatment with a GLP-1 RA. Recommended management includes immediate discontinuation of the medication, patient counseling with appropriate reassurance, initiation of standard prenatal care, and ongoing obstetric and metabolic monitoring. Current human data do not demonstrate a consistent increase in major congenital malformations with inadvertent early-pregnancy exposure. However, evidence remains limited, and animal studies have raised safety concerns, particularly regarding maternal weight loss. Data are derived from current clinical guidelines, prescribing information, and systematic reviews of animal and human pregnancy outcomes [3,14,18-20,22]. GLP-1 RA: glucagon-like peptide-1 receptor agonist

For patients in whom pregnancy is recognized during GLP-1 RA therapy, a management pathway for inadvertent pregnancy exposure is shown in Figure 3.

Key elements of periconception counseling should include the following: (1) Contraception: Reproductive-aged patients using GLP-1 RAs who are not planning pregnancy should be counseled about effective contraception, including those with a history of infertility, because improved metabolic status and weight loss may improve ovulatory function. Long-acting reversible contraception may be preferred for patients who desire it, particularly for those using tirzepatide, because these methods avoid potential oral contraceptive absorption concerns [3,4,16,30].

(2) Planned discontinuation: For patients planning pregnancy, semaglutide should be stopped at least two months before conception [18], and tirzepatide should be stopped at least one month before conception according to Canadian labeling [29]. The washout period should also allow time for medication elimination, transition to pregnancy-compatible therapies, and achievement of preconception glycemic goals [22].

(3) Metabolic rebound counseling: Patients should be informed about the risk of weight regain and worsening glycemic control after GLP-1 RA discontinuation. Monitoring, nutritional counseling, and lifestyle support should be initiated when therapy is stopped [3,20].

(4) Inadvertent exposure: If pregnancy is recognized while using a GLP-1 RA, the medication should generally be discontinued, and the patient should be counseled promptly. Current human evidence does not indicate that inadvertent early-pregnancy exposure warrants undue alarm, but individualized obstetric follow-up and metabolic monitoring are appropriate [4,20].

Use during pregnancy: current evidence and guidelines

GLP-1 RAs are not recommended for routine use during pregnancy in current product labeling and professional guidance [3,18,22]. FDA labeling for semaglutide states that fetal harm may occur based on animal reproduction studies, while available human data remain insufficient to establish a drug-associated risk [14,18]. A pregnancy exposure registry has also been established to monitor outcomes among patients exposed to semaglutide during pregnancy [18].

Although these medications are generally stopped before conception, pregnancy exposure still occurs in clinical practice. One retrospective cohort study using linked electronic medical record and pharmacy data evaluated pregnancy outcomes among patients with overweight or obesity who had semaglutide exposure [23]. Among 429 pregnancy-exposed users, the median duration of exposure during pregnancy was 44 days [23]. Compared with nonusers, exposed patients had higher adjusted odds of excessive gestational weight gain, gestational diabetes, excessive fetal growth, and cesarean delivery [23]. However, similar risk elevations were also seen among former users who stopped semaglutide before pregnancy, suggesting that the findings may reflect baseline cardiometabolic risk, rebound after discontinuation, or residual confounding rather than a direct intrauterine drug effect [23].

A large matched observational cohort study also found that GLP-1 RA exposure followed by discontinuation before pregnancy or early in pregnancy was associated with approximately 3 kg greater gestational weight gain and increased risks of excess gestational weight gain, preterm delivery, gestational diabetes, and hypertensive disorders of pregnancy compared with unexposed individuals [25]. These data suggest that metabolic changes after discontinuation may require close monitoring, although causality cannot be inferred from observational evidence.

Other studies have reported conflicting findings. One retrospective analysis reported lower odds of hypertensive disorders of pregnancy among patients with peripregnancy GLP-1 RA exposure, including both patients with pregestational diabetes and those using these medications for weight management [26]. These associations were slightly stronger among patients exposed during pregnancy [26]. Such findings highlight the potential for confounding by indication, baseline metabolic status, timing of medication discontinuation, and differences in clinical follow-up.

GLP-1 RAs have been identified in the human placenta, but available evidence suggests limited placental transfer for some GLP-1 RAs [3,19]. The clinical relevance of placental GLP-1 RA signaling during medication exposure remains uncertain [3].

Overall, GLP-1 RAs should not be intentionally used during pregnancy outside of exceptional circumstances or research settings. If exposure occurs before pregnancy recognition, available human data do not clearly indicate a major teratogenic signal; however, the medication should generally be discontinued, and the patient should receive individualized obstetric and metabolic follow-up.

Postpartum and lactation considerations

Postpartum Use

The postpartum period has become an increasingly common window for GLP-1 RA prescribing. In Denmark, postpartum use of GLP-1 RAs increased from fewer than five users per 10,000 births in 2018 to 173 per 10,000 births by mid-2024, driven largely by semaglutide prescriptions for weight loss [27]. A similar pattern has been reported in the United States, where postpartum prescribing increased from 0.3 to 14.6 per 1,000 deliveries between 2019 and 2024 [2]. Most postpartum users in the Danish cohort had overweight before pregnancy, and only 23% had a documented diabetes diagnosis, suggesting that weight reduction was the primary indication for many patients [27].

The American College of Cardiology Expert Consensus Decision Pathway acknowledges the potential role of GLP-1 RAs for postpartum weight management but notes that lactation safety data remain limited [28]. If GLP-1 RA therapy is considered during lactation, injectable formulations are generally preferred over oral semaglutide due to concerns about oral formulation components and limited breastfeeding data [28]. Semaglutide labeling states that the effect of semaglutide on breastfed infants is unknown, while lactation-specific recommendations vary by formulation [18].

Lactation Safety

Evidence on GLP-1 RA transfer into human milk remains sparse. Animal studies have shown drug excretion into milk, but the relevance of these findings to human lactation is uncertain [19]. In one small pharmacokinetic study involving eight lactating women, semaglutide was not detected in human milk, and the estimated relative infant dose was far below commonly used safety thresholds [20]. However, this evidence is limited because the study evaluated only one medication, had a small sample size, used limited sampling, did not measure infant serum concentrations, and had limited follow-up of breastfed infants [20].

FDA labeling states that the effect of semaglutide on breastfed infants is unknown [18]. Given the large molecular size and peptide-based structure of GLP-1 RAs, significant oral bioavailability in infants is considered unlikely, though this has not been confirmed in larger clinical studies [19].

Clinicians should use shared decision-making with postpartum patients, weighing the potential benefits of weight management and metabolic improvement against the limited safety data during lactation. For patients who choose to breastfeed, the decision to initiate GLP-1 RA therapy should be individualized, with consideration of infant age, degree of breastfeeding, maternal cardiometabolic risk, and the availability of alternative weight-management strategies.

Gestational weight management and implications for future pregnancies

A key concern surrounding GLP-1 RA use in the periconceptional period is metabolic rebound after discontinuation. In nonpregnant adults, discontinuation of GLP-1 RAs has been associated with substantial weight regain [3]. When discontinuation occurs before conception or during early pregnancy, rebound weight gain or worsening glycemic control may contribute to excessive gestational weight gain and related pregnancy complications, although causality remains uncertain.

Multiple studies have described this pattern. In a large matched cohort study, patients who stopped GLP-1 RAs before pregnancy or during early pregnancy gained approximately 3 kg more during gestation than matched unexposed controls and had a 32% higher risk of excessive gestational weight gain [25]. A retrospective study reported that semaglutide-exposed users had nearly three-fold higher odds of excessive gestational weight gain compared with nonusers [23]. Case reports have also described marked rebound weight gain after semaglutide cessation during pregnancy, including increases of up to 35 kg [20].

However, the literature is not entirely consistent. In one randomized controlled trial of women with overweight or obesity and PCOS, preconception exenatide pretreatment was followed by gestational weight gain similar to that seen with metformin, with mean gains of 9.62 kg and 9.81 kg, respectively [20]. A single-center retrospective case-control study also reported lower gestational weight gain among women with prior GLP-1 RA exposure compared with controls [20].

These mixed findings likely reflect differences in baseline metabolic risk, indication for treatment, timing of medication discontinuation, magnitude of post-cessation weight rebound, and intensity of pregnancy monitoring rather than a direct pharmacologic effect alone [20]. Clinically, patients discontinuing GLP-1 RAs in preparation for pregnancy may benefit from close monitoring of weight trajectory, nutritional counseling, glycemic surveillance when indicated, and lifestyle support to mitigate rebound weight gain.

For future pregnancies, the interpregnancy interval is an important window for optimizing weight. Interpregnancy weight loss has been associated with reduced risk of subsequent adverse pregnancy outcomes, including gestational diabetes and preeclampsia [28]. GLP-1 RAs may have a role in interpregnancy weight management, but timing should be coordinated with breastfeeding goals, medication washout recommendations, and plans for subsequent conception.

Knowledge gaps and future research directions

Despite rapidly accumulating evidence, important knowledge gaps remain regarding the use of GLP-1 RAs across the reproductive lifespan. Current human data are largely observational and limited by small exposed sample sizes, heterogeneous patient populations, variable exposure windows, incomplete medication-discontinuation data, and limited information on glycemic control, weight trajectory, and long-term offspring outcomes [3,20].

Pregnancy Safety

Prospective, adequately powered studies are needed to evaluate the safety of GLP-1 RA exposure during pregnancy. The Endocrine Society has proposed several priority research areas, including assessment of fetal development, congenital anomalies, spontaneous abortion, fetal growth, and preterm birth after GLP-1 RA exposure or discontinuation [3]. Further studies should also compare short-acting and long-acting GLP-1 RAs in the preconception period, since drug persistence and washout timing differ by agent. Pregnancy exposure registries will be important for capturing outcomes among patients who unintentionally continue these medications during pregnancy or become pregnant before washout is complete [3].

Lactation

Lactation data remain particularly limited [20]. Only one structured pharmacokinetic study has evaluated semaglutide transfer into human milk, and available studies have not adequately assessed infant outcomes following maternal use of GLP-1 RAs during breastfeeding [20]. Data regarding tirzepatide, liraglutide, dulaglutide, and other GLP-1-based therapies during lactation remain sparse.

Contraceptive Interactions

Although the interaction between tirzepatide and oral hormonal contraceptives has been characterized pharmacokinetically, its clinical significance in real-world settings remains unknown, including whether it affects unintended pregnancy rates [16]. Additional studies are needed to evaluate whether newer oral GLP-1 RA formulations affect oral contraceptive absorption and contraceptive efficacy.

Long-Term Offspring Outcomes

Follow-up data for children exposed to GLP-1 RAs in utero remain very limited. Available case reports have described reassuring early growth and neurodevelopment through approximately 12-14 months. Still, these observations are based on small sample sizes and lack standardized neurocognitive assessments or comparison groups [20]. Longer-term studies are needed to determine whether exposure during pregnancy or lactation affects metabolic, developmental, or neurocognitive outcomes in offspring.

Metabolic Rebound

The mechanisms underlying post-discontinuation metabolic rebound and its specific impact on pregnancy physiology require further study. It remains unclear whether strategies such as gradual dose tapering, transition to alternative therapies, structured lifestyle intervention, or closer metabolic monitoring can reduce rebound weight gain or worsening glycemic control during the periconceptional period.

Equity and Access

As GLP-1 RA use expands, disparities in access, counseling, affordability, and clinical monitoring may disproportionately affect vulnerable populations. Future research should evaluate whether the benefits and risks of periconceptional GLP-1 RA use differ across racial, ethnic, socioeconomic, and geographic groups.

Overall Evidence Limitations and Counseling Implications

Despite increasing clinical use of GLP-1 RAs and dual GIP/GLP-1 RAs among reproductive-aged women, evidence guiding reproductive counseling remains limited. Current recommendations are based largely on preclinical studies, pharmacokinetic analyses, observational cohorts, regulatory labeling, pregnancy exposure reports, and expert guidance rather than prospective trials specifically designed to evaluate reproductive, pregnancy, and lactation outcomes. Counseling should therefore remain individualized, agent-specific, and updated as additional pregnancy registry, pharmacovigilance, and post-marketing safety data become available.

## Conclusions

GLP-1 RAs and dual GIP/GLP-1 RAs have important implications for reproductive health as their use expands among reproductive-aged patients. Current evidence suggests potential benefits for ovulatory function in some patients with PCOS, but this also increases the importance of proactive contraception and preconception counseling. Tirzepatide warrants particular attention because it may reduce oral contraceptive exposure during initiation and dose escalation, requiring backup or non-oral contraception during these periods.

Animal reproductive studies continue to justify caution, whereas currently available human data have not shown a consistent major teratogenic signal after inadvertent early-pregnancy exposure. Product labeling and professional guidance continue to recommend discontinuation before pregnancy, including a two-month washout for semaglutide before planned conception. Patients discontinuing therapy should be counseled about possible weight regain, worsening glycemic control, and the need for nutritional, metabolic, and obstetric monitoring.

Postpartum prescribing is increasing, but lactation-specific safety data remain limited. Counseling should therefore remain individualized, agent-specific, and guided by pregnancy intention, contraceptive reliability, metabolic risk, breastfeeding goals, and shared decision-making. Larger prospective studies, pregnancy exposure registries, and long-term follow-up of exposed children are needed to clarify reproductive, pregnancy, and lactation safety.
